# The Effect of Pain Catastrophizing on Depression among Older Korean Adults with Chronic Pain: The Mediating Role of Chronic Pain Interference and Sleep Quality

**DOI:** 10.3390/ijerph17238716

**Published:** 2020-11-24

**Authors:** Kyoung-eun Lee, Hyunju Ryu, Sun Ju Chang

**Affiliations:** 1 College of Nursing, Seoul National University, 103 Daehak-ro, Jongno-gu, Seoul 03080, Korea; jitta123@snu.ac.kr (K.-e.L.); rhj1110@snu.ac.kr (H.R.); 2 The Research Institute of Nursing Science, Seoul National University, 103 Daehak-ro, Jongno-gu, Seoul 03080, Korea

**Keywords:** aged, depression, pain catastrophizing, chronic pain, sleep quality

## Abstract

Pain catastrophizing is a notable concept associated with change in chronic pain interference and depression. Sleep quality is also one of the important factors affecting geriatric depression. This study examined the mediating effects of chronic pain interference and sleep quality on the relationship between pain catastrophizing and depression. This study is a secondary data analysis that analyzed a total of 138 older Korean adults with chronic pain. The participants were selected from a single elderly daycare center in a city in South Korea. Also, the multiple regression analysis and PROCESS macro with bootstrapping were used. The results revealed that chronic pain interference and sleep quality mediated the relationship between pain catastrophizing and depression, respectively. Furthermore, chronic pain interference and sleep quality sequentially and dually mediated the effect of pain catastrophizing on depression. In the management of depression in the elderly, persistent complaints of pain should not be disregarded, irrespective of the intensity of their chronic pain. Psychological intervention is needed to alleviate negative thoughts about chronic pain and to increase the ability to cope with chronic pain. In addition, it is important to assess sleep patterns and to develop interventions to improve sleep quality, because depression in the elderly could appear as a symptom of a sleep problems.

## 1. Introduction

Korea is classified as a country where the elderly population is growing rapidly, with the proportion of older adults (age ≥ 65 years) in the general population standing at 14.9% [1,2]. Accordingly, there is a need for nurses to pay attention to the health problems and quality of life of older adults.

Many older adults experience mental health problems such as feelings of loss and fear of death, as well as social problems such as recent bereavement, and worsening economic conditions [3]. They also often experience a deterioration in their health as a result of the pathological processes of aging and physical illness [2]. Depression caused by these psychosocial and physical exacerbations is the most common psychiatric problem in old age. Depression in the elderly is defined as depression affecting those above the age of 65 years [4]. In Korea, as of 2017, the prevalence of depression in the elderly (21.1%) is more than four times higher than the prevalence of depression in the population under 64 (5.1%), indicating that this is a very serious problem for the elderly [5]. In general, risk factors for depression in old age can be divided into biological factors such as physical illness, drugs, and alcohol, and psychosocial factors such as low economic status, stress, lack of social support, and pain [2].

In particular, depression in the elderly is closely associated with chronic pain. According to the International Association for the Study of Pain, pain is defined as actual or potential tissue damage, or the unpleasant sensations and emotional experiences associated with it [6]. Pain is determined by the patient’s personal judgment and is one of the major health problems that affect older adults. Most older adults live with moderate-intensity pain, and the pain duration of more than 50.0% of older adults is longer than 3 years [7]. Higher levels of chronic pain are associated with higher levels of depression and suicide intentions among adults [8]. Especially, a cohort study found that elderly patients lose control of their lives due to pain interference, which increases their depressive symptoms [9]. Robinson’s study shows that the intensity of chronic pain is not associated with pain relief, and that a predictor of depression is pain interference [10]. In other words, chronic pain cannot be explained only by pain intensity [11,12], and the evaluation of pain interference is an important component in chronic pain measurement and treatment [13].

Recently, a notable concept related to depression is pain catastrophizing [14,15,16], defined as sustained negative thoughts resulting from an excessive fear of pain and consisting of three subfactors: rumination, magnification, and helplessness [17,18]. Furthermore, pain catastrophizing was found to be a prediction factor not only for depression but also for chronic pain interference [12]. Thus, pain catastrophizing is considered an important variable in understanding the psychological mechanism in depression with chronic pain.

Meanwhile, sleep quality is one of the factors that strongly affects geriatric depression [19] and is also associated with pain catastrophizing [20,21]. Senba’s study found the general concept that insomnia, chronic pain, and depression are mutually interacting [22]. Also, sleep disturbance is reported to be correlated with pain interference [23].

**Figure 1:**
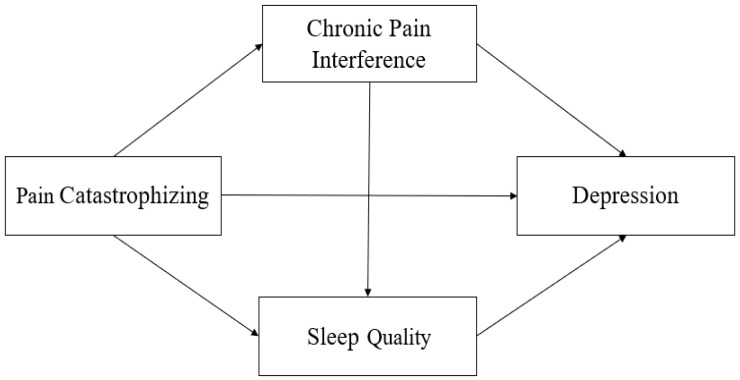
The hypothetical model.

As indicated above, pain catastrophizing, chronic pain interference, and sleep quality have been identified as important factors related to geriatric depression. In summary, chronic pain has a psychological aspect as a personal experience that cannot be defined only as actual tissue damage [6]. Pain catastrophizing, sleep quality, and depression also have psychological and cognitive characteristics [12,24], and they are closely related. Based on the results of previous studies [19,21,25], we developed the hypothetical model (Figure 1) that assumes a mediating role of chronic pain interference and sleep quality in the relationship between pain catastrophe and depression in the elderly.

The aims of this study are: (1) to examine the relationships between pain catastrophizing, chronic pain interference, sleep quality, and depression; (2) to analyze the multiple mediating effects of chronic pain interference and sleep quality between pain catastrophizing and depression.

## 2. Methods

### 2.1. Study Design

Using secondary data, we examined the mediating effects of chronic pain interference and sleep quality on the relationship between pain catastrophizing and depression among older Korean adults with chronic pain. In the parent study [26], the factors that influence chronic pain among Korean and Korean-American older adults were compared. In this study, only the data collected from older Korean adults were analyzed.

### 2.2. Participants

The target population of the parent study comprised older Korean adults with chronic pain [26]. Convenience sampling was used to recruit participants from a single elderly daycare center in a city in South Korea. The sample inclusion criteria were as follows: age ≥ 65 years, has had chronic pain for the previous three months, and does not have any cognitive impairments that may interfere with his or her participation in the study (i.e., Korean Mini-Mental Status Examination: score > 24). A total of 138 older Korean adults voluntarily consented to participate in the parent study [26]. Post-hoc power analysis was conducted using G*power 3.1 (Düsseldorf, Germany) and the following specifications: effect size = 0.15, significance level = 0.05, total sample size = 138. The computed power was over 0.97. This indicated that the sample size (i.e., 138) was adequate for the present analysis.

### 2.3. Measurements

#### 2.3.1. Demographic and Disease-Related Characteristics

Demographic characteristics of age, gender, educational level, marital status, socioeconomic status, comorbidities and chronic pain areas were recorded.

#### 2.3.2. Depression

The Patient Health Questionnaire-9 Korean Version (PHQ-9K) was used to assess depression. The PHQ-9K is Park’s Korean adaptation of a screening tool for depression that was originally developed by Spitzer, Kroenke, and Williams [27,28]. This self-administered questionnaire consists of nine items that assess the nine diagnostic criteria for major depressive syndrome. It assesses the frequency with which a respondent has been experiencing depressive symptoms during the past two weeks. Responses are recorded on a scale that ranges from 0 to 3 (0 = not at all, 1 = several days, 2 = more than half the days, 3 = almost every day). Total scores served as a measure of depressive symptoms, with higher scores indicative of a higher level of depression. In the present study, we calculated the total score of the 8 items excluding one item related to sleep problems. The alpha coefficient of this scale was 0.84 in Park’s study [27] and 0.85 in the current study.

#### 2.3.3. Pain Catastrophizing

The Pain Catastrophizing Scale is a measure of pain catastrophizing [18]. This 13-item scale assesses the extent to which a respondent engages in catastrophic thinking in response to pain and his or her pain-related cognitive-emotional processes by requiring him or her to recall past experiences of pain. A composite score can be computed by adding the scores of the 13 individual items, with higher scores indicative of a greater degree of pain catastrophizing. The Korean version of this assessment that has been validated among patients with chronic non-cancer pain was used in this study [29]. The alpha coefficient of this scale was 0.93 in both the study in which the Korean version was validated and present study.

#### 2.3.4. Sleep Quality

Sleep quality was assessed using the Pittsburgh Sleep Quality Index [30]. This assessment consists of 19 self-report and five other-report items. In this study, only the 19 self-report items were used. Higher scores are indicative of poorer sleep quality. The Korean version of this assessment was developed and validated by Sohn [31]. The alpha coefficient of this scale was 0.84 in the study in which the Korean version was validated and 0.70 in the present study.

#### 2.3.5. Chronic Pain Interference

We measured chronic pain interference using the pain interference scale of the Brief Pain Inventory Short Form [32]. The Brief Pain Inventory (BPI) assesses both the intensity of pain (i.e., sensory dimension of pain) and the degree to which pain interferes (i.e., reactive dimension of pain) with functioning [32]. The pain interference scale consists of seven items, and evaluates pain interference (i.e., the effect of pain on function) in a patient’s general activity, mood, walking ability, work, interpersonal relationships, sleep, and enjoyment of life during the last 24 h. Responses are recorded on an 11-point scale (0 = no interference, 10 = complete interference). A composite score can be computed by averaging the individual scores of the seven items. In the present study, we calculated the total score of the six items excluding one item related to sleep. Higher scores are indicative of greater effects of pain. The Korean version of the BPI (BPI-K) was used in the present study [33]. In the study in which the Korean version of this assessment was validated, the Cronbach’s alpha of the pain intensity and interference subscales were 0.85 and 0.93, respectively [33]. In the current study, the Cronbach’s alphas of pain interference subscales were 0.92.

### 2.4. Data Collection

Data collection of the parent study was conducted in an elderly daycare center in a city in South Korea, and collected between January and February 2019 [26]. All researchers in this study conducted the survey face-to-face with the participants. We provided a self-report assessment to those who voluntarily participated in the study and provided written consent. The average time required to complete the assessments was 30 min. Once participants had completed the assessments, one of the researchers immediately inputted the collected data into a database.

### 2.5. Ethical Considerations

The parent study was conducted with the approval of the institutional review board (IRB) of Seoul National University to which the researchers were affiliated (IRB No. 1808/002-009) [26]. In this study, the data were analyzed only after an exemption from review was approved by the IRB of the same university (IRB No. E1908/001-005). Eligible participants (i.e., based on the inclusion and exclusion criteria) who understood the purpose of the study and were willing to participate on a voluntary basis were recruited. Before the participants provided signed informed consent, they were provided with an explanatory manual that contained information about the following: the need for the study, study objectives, potential benefits of participation, data collection methods, the time that they would be required to invest in the study, and the researcher’s contact information. It also assured them of the confidentiality of their data and anonymity of their participation.

### 2.6. Data Analysis

The collected data were analyzed using SPSS version 23.0 (IBM, New York, NY, USA). First, participants’ demographic and disease and pain related characteristics were examined by computing descriptive statistics. Second, the relationships between pain catastrophizing, chronic pain interference, sleep quality, and depression were analyzed using descriptive statistics, correlation analysis, and multiple regression analysis. Finally, to analyze the mediating effect of chronic pain interference and sleep quality on the relationship between pain catastrophizing and depression, the PROCESS macro and bootstrapping (PROCESS v3.3 by Andrew F. Hayes, Columbus, OH, USA) were used. This method complements the limited power of the Sobel test, which relies on the assumption of normality. In this study, 10,000 bootstrap samples were used. The mediating effect was considered to be significant at *p* < 0.05, if a 0 was not included in the 95% bootstrap confidence intervals for indirect effects.

## 3. Results

### 3.1. Demographic and Disease and Pain Related Characteristics

Participants’ demographic characteristics are shown in Table 1. The sample (*N* = 138) consisted of 61 men (44.2%) and 77 women (55.8%), with a mean age of 75.44 years (95% CI: 74.31–76.58). A total of 34 (24.6%) participants were found to have graduated from middle school, and 66 (47.8%) respondents indicated that their financial resources were rather inadequate to meet their daily needs. Moreover, 73 respondents (52.9%) were married, and 121 participants (87.7%) had a disease, most commonly hypertension (*N* = 64, 46.4%), followed by arthritis (36.2%) and eye diseases (26.1%). Participants complained of chronic pain in various body parts. A total of 49 participants (35.5%) had knee pain, 28 (20.3%) complained of shoulder pain, and 22 (15.9%) had spinal pain. Further, 16 participants (11.6%) had pain in the hip part including the hip joint.

### 3.2. Characteristics and Correlation of Pain Catastrophizing, Chronic Pain Interference, Sleep Quality, and Depression

Descriptive statistics and correlation analysis of variables are shown in Table 2. The mean score of each variable was as follows: Pain catastrophizing was 13.48 (95% CI: 11.63–15.31), chronic pain interference was 2.49 (95% CI: 2.13–2.85), sleep quality was 6.03 (95% CI: 5.41–6.66), and depression was 3.69 (95% CI: 3.00–4.40). Skewness and kurtosis were used to evaluate the normality of the scale. Normal distribution is assumed when the absolute values of kurtosis and skewness are less than 3.0. The skewness and kurtosis of the variables used in this study were all less than 3.0, and a normal distribution can be assumed. In addition, depression showed a statistically significant correlation with pain catastrophizing (r = 0.38, *p* < 0.01), chronic pain interference (r = 0.45, *p* < 0.01), and sleep quality (r = 0.34, *p* < 0.01).

### 3.3. The Relationships between Pain Catastrophizing, Chronic Pain Interference, Sleep Quality, and Depression

To examine the relationships between the four variables, the effects of pain catastrophizing, chronic pain interference, and sleep quality on depression were analyzed using a multiple regression analysis. Before the multiple regression analysis was conducted, pertinent assumptions were tested: the assumptions of autocorrelation (i.e., within dependent variables) and multicollinearity (i.e., between independent variable). The Durbin-Watson statistic was found to be 1.988, which is lower than 2.233 (*d_u_* < *d* < 4 − *d_u_*). This was indicative of a relative absence of autocorrelations. Also, the Variance Inflation Factor (VIF) values (range = 1.118–1.377) were lower than 10; this indicated that there was no multicollinearity. Thus, all the assumptions of regression analysis were satisfied. As shown in Table 3, chronic pain interference (*p* < 0.001) and sleep quality (*p* < 0.05) had a statistically significant effect on depression. In other words, statistically, the higher the level of chronic pain interference (B = 0.641) and the lower the quality of sleep (B = 0.241), the higher the depression. However, pain catastrophizing did not have a statistically significant effect on depression. The explanatory power of this regression model was 25.0%.

### 3.4. The Mediating Effects of Chronic Pain Interference and Sleep Quality

The results of the mediating effects of chronic pain interference and sleep quality are presented in Table 4. Chronic pain interference mediated (B = 0.063) the relationship between pain catastrophizing and depression. The results showed that sleep quality also mediated (B = 0.017) this relationship. Moreover, chronic pain interference and sleep quality were found to have a sequential and dual mediating effect (B = 0.006) in the relationship between pain catastrophizing and depression. Finally, the total effect of pain catastrophizing on depression was significant (C = 0.132, *p* < 0.001).

## 4. Discussion

Depression in the elderly is a serious disease, and its prevalence is four times higher among older adults than among younger adults. Multiple nursing approaches should be adopted to examine geriatric depression (both its physical and psychological aspects), and in-depth analyses of influential factors should be undertaken. Accordingly, this study aimed to examine the mediating effects of chronic pain interference and sleep quality on the effects of pain catastrophizing on depression among older adults with chronic pain.

Overall, pain catastrophizing (i.e., independent variable) did not have a direct effect on depression (i.e., dependent variable), but the total effect of this hypothetical model was significant. The total effects were the sum of the direct and indirect effects. This indicates that pain catastrophizing had an indirect effect on depression through chronic pain or sleep quality. The question of whether pain catastrophizing has a direct effect on depression continues to be debated. The results of Paré et al.’s study, which showed high correlations between pain catastrophizing and depression [34] and Nicholas et al.’s study, which found that depression correlated more with cognitive variables than pain itself, underscored the direct effects of pain catastrophizing [35]. In contrast, Hülsebusch et al. found that pain catastrophizing had an indirect effect on depression through helplessness/hopelessness, and this observation is similar to the findings of this study [36].

With regard to the studies that have demonstrated support for the direct effects of pain catastrophizing on depression, this point is noteworthy: the sample consisted only of those with specific diseases or pain areas [14,35,37]. In the present sample, there were greater variability in the diseases that caused chronic pain and chronic pain areas. Thus, greater diversity of chronic pain experiences among the participants may have influenced the relationship between pain catastrophizing and depression. As a result, pain catastrophizing may not have had a direct effect on depression.

Next, we examined the indirect effects of pain catastrophizing on depression through chronic pain interference and sleep quality. First, when we analyzed the effect of pain catastrophizing on depression though chronic pain interference (i.e., mediating variable), we found that chronic pain interference had a significant mediating effect. Zis et al. found that chronic pain increases the risk of depression, and the degree of pain perception increases the association with depression among the elderly with chronic pain [6]. Chronic pain interference reflects patients’ perception of the extent to which pain interferes with social relationship, work or daily activities [38]. Rapti et al.’s study also showed that the higher the pain interference, the greater the symptoms of depression [39].

Meanwhile, Edwards et al. found that patients with chronic pain spend a lot of their time reflecting on their pain and that this preoccupation contributes to the recurrence and persistence of pain [40]. Noyman-Veksler et al. explained that a high level of pain catastrophizing ultimately exacerbates the patient’s pain, and these patients excessively focus on pain, resulting in more depressive symptoms [41]. In other words, older adults with high levels of pain catastrophizing tend to ruminate about their pain, which contributes to its recurrence and persistence. Pain catastrophizing can also sustain pain and disrupts daily life by making them pay undue attention to the negative aspects of chronic pain. Consequently, a higher pain interference exacerbates depression by losing control of life. In addition, from a physiological point of view, it has been noted that the nociceptive and emotion pathways are anatomically consistent. The biochemical theory of depression assumes that depression is the result of a functional deficiency of norepinephrine and serotonin, and deficiency of these neurotransmitters affects pain development [42,43]. Therefore, with regard to the management of depression in older adults with chronic pain, persistent complaints of pain should not be disregarded, irrespective of the intensity of their pain. Instead, pain management methods should be used to relieve pain and reduce the sensitivity of pain perception. In addition to using pharmacological pain management methods (i.e., medication) to directly treat pain, psychosocial interventions should be provided to address their maladaptive thoughts about chronic pain and their ability to cope with pain.

Second, sleep quality had a mediating role on the effect of pain catastrophizing on depression. Many studies have found that sleep quality and depression are strongly intercorrelated. Gee et al. found that poor sleep can cause depression [44]. Additionally, O’Leary and Lee found that sleep disorders are predictors of depression; these findings are similar to the results of this study [45,46]. Sleep problems increase the risk of depression among patients with pain [47]. Older adults who engage in pain catastrophizing experience pain magnification, rumination, and helplessness, and these cognitive distortions can adversely affect sleep quality and cause insomnia. Such sleep disorders can cause older adults to stay in bed for longer durations during the daytime. As a result, they may not be motivated to engage in daily life activities [48]. Withdrawal and a failure to engage in daily life activities can further exacerbate depression [49]. Thus, to manage depression among older adults with chronic pain, it is important to assess their sleep patterns. In particular, Chun found that depression in older adults can easily be overlooked because it can appear to be a symptom of insomnia rather than depression [50]. Also, sleep disturbance is the most common symptom of depression [51]. Accordingly, when working with older adults who frequently complain of pain, it is necessary to prove their sleep quality.

Finally, we found that chronic pain interference and sleep quality sequentially and dually mediated the effects of pain catastrophizing on depression. These findings suggest that pain catastrophizing does not have a direct effect on depression among older adults with chronic pain. Instead, pain catastrophizing has an effect on chronic pain interference, and increased chronic pain interference result in poorer sleep quality, which in turn has an effect on depression. Chronic pain interference leads to a loss of control over the elderly’s lives [9] and prevents older adults from procuring sufficient rest and sleep [47]. Chronic pain interferes with sleep, reduces activity levels, and lowers mood [52]. The last path that emerged suggests that chronic pain interference and sleep quality are vital factors in the management of depression among older adults. Accordingly, nursing intervention programs to manage chronic pain interference and sleep quality are likely to be highly effective in preventing depression.

Despite its significant contributions, this study has some limitations. First, in the parent study, convenience sampling was used to recruit older adults with chronic pain from only one seniors’ welfare center. Thus, because of their unique regional characteristics, the findings may not be generalizable to all older adults. In addition, secondary data were analyzed. Since the variables were predetermined, the results may have been biased.

## 5. Conclusions

We found that pain catastrophizing has no direct effect on depression. However, pain catastrophizing was mediated by chronic pain interference and sleep quality, which had a statistically significant effect on depression. Further, chronic pain interference and sleep quality were sequential dual mediators in the relationship between pain catastrophizing and depression. Based on the present findings, we offer four recommendations. First, healthcare providers should possess an in-depth understanding of the factors affecting geriatric depression and the interrelationships among them. Further, they should be cognizant of these factors when attempting to treat geriatric depression. Most importantly, chronic pain and sleep quality should be routinely assessed to screen for depression among older adults. Second, to prevent depression among older adults, it is necessary to develop nursing interventions that address pertinent psychosocial factors (e.g., pain catastrophizing, sleep quality). Third, expanding on the relationships found in this study, future studies may consider a pass analysis or a structural equation model for geriatric depression. Finally, since various interrelationships may exist between the variables of this study, future studies using longitudinal data are required.

## Figures and Tables

**Table 1:** General characteristics of the participants (*N* = 138).

Characteristics	*n* (%)	Mean (95% CI)
**Gender**		
Male	61 (44.2)	
Female	77 (55.8)	
**Age (years)**		75.44 (74.31–76.58)
**Educational level**		
No Education	2 (1.4)	
Elementary school	30 (21.7)	
Middle school	34 (24.6)	
High school	31 (22.5)	
Diploma	12 (8.7)	
Bachelor’s	29 (21.0)	
**Perceived socioeconomic status**		
Very difficult for daily living	16 (11.6)	
Somewhat difficult for daily living	66 (47.8)	
Not difficult for daily living	56 (40.6)	
**Marital status**		
Married/partnered	73 (52.9)	
Single	65 (47.1)	
**Current diagnosed disease**		
Yes	121 (87.7)	
No	17 (12.3)	
**Type of diseases (multiple responses)**		
Hypertension	64 (46.4)	
Arthritis	50 (36.2)	
Eye related diseases	36 (26.1)	
Diabetes	28 (20.3)	
Osteoporosis	23 (16.7)	
Prostate disease	22 (15.9)	
**Type of Chronic pain area (multiple responses)**		
Knee	49 (35.5)	
Shoulder	28 (20.3)	
Spine	22 (15.9)	
Hip (Including Hip Joint)	16 (11.6)	
Arm (Including elbow)	13 (9.4)	
Thigh	10 (7.2)	
Foot (Including Ankle)	9 (6.5)	
Stomach	7 (5.1)	
Back	6 (4.3)	
Chest	6 (4.3)	
Neck	6 (4.3)	
Others (Head, mouth, hand, ear, face, eye)	16 (11.6)	

**Table 2:** Descriptive statistics and correlation of variables.

Variable	Pain Catastrophizing	Chronic Pain Interference	Sleep Quality	Mean	95% CI
Pain catastrophizing				13.48	11.63–15.31
Chronic pain interference	0.50 **			2.49	2.13–2.85
Sleep quality	0.31 **	0.25 **		6.03	5.41–6.66
Depression	0.38 **	0.45 **	0.34 **	3.69	3.00–4.40

** *p* < 0.01, CI: Confidence intervals.

**Table 3:** Effects of Pain Catastrophizing, Chronic Pain Interference, and Sleep Quality on Depression.

Variable	B	SE	β	t	*P*
(Constant)	−0.018	0.683		−0.158	0.875
Pain catastrophizing	0.046	0.033	0.125	1.375	0.172
Chronic pain interference	0.641	0.169	0.339	3.798	0.000
Sleep quality	0.241	0.094	0.210	2.575	0.011

*_adj_R^2^* = 0.250, F = 15.004 (*p* < 0.001), Durbin-Watson = 1.988 (*d_u_* = 1.767).

**Table 4:** The Mediating effects of Chronic Pain interference and Sleep Quality in the Relationship between Pain Catastrophizing and Depression.

Mediating Path	Mediating Effect		
	B	SE	95% CI
Pain catastrophizing→Chronic pain interference→Depression	0.063	0.018	0.032–0.103
Pain catastrophizing→Sleep quality→Depression	0.017	0.012	0.000–0.044
Pain catastrophizing→Chronic pain interference→Sleep quality→Depression	0.006	0.005	0.000–0.020

C = 0.132 (*p* < 0.001).
